# Biocompatible α‐Methylenation of Metabolic Butyraldehyde in Living Bacteria

**DOI:** 10.1002/anie.202306347

**Published:** 2023-08-09

**Authors:** Jonathan A. Dennis, Nick W. Johnson, Thomas W. Thorpe, Stephen Wallace

**Affiliations:** ^1^ Institute of Quantitative Biology, Biochemistry and Biotechnology, School of Biological Sciences University of Edinburgh Edinburgh EH9 3FF UK; ^2^ EaStCHEM School of Chemistry University of Edinburgh Edinburgh EH9 3FJ UK

**Keywords:** Biocompatible Chemistry, Biotechnology, Green Chemistry, Organocatalysis

## Abstract

Small molecule organocatalysts are abundant in all living organisms. However, their use as organocatalysts in cells has been underexplored. Herein, we report that organocatalytic aldol chemistry can be interfaced with living *Escherichia coli* to enable the α‐methylenation of cellular aldehydes using biogenic amines such as L‐Pro or phosphate. The biocompatible reaction is mild and can be interfaced with butyraldehyde generated from D‐glucose via engineered metabolism to enable the production of 2‐methylenebutanal (2‐MB) and 2‐methylbutanal (2‐MBA) by anaerobic fermentation, and 2‐methylbutanol (2‐MBO) by whole‐cell catalysis. Overall, this study demonstrates the combination of non‐enzymatic organocatalytic and metabolic reactions in vivo for the sustainable synthesis of valuable non‐natural chemicals that cannot be accessed using enzymatic chemistry alone.

## Graphical abstract

Introducing new abiotic chemistry into living cells is a grand challenge in the field of chemical biotechnology. Here, we show that phosphate and/or biogenic amines mediate the non‐enzymatic α‐methylenation of butyraldehyde under biocompatible reaction conditions and can be interfaced with butyraldehyde biosynthesis from D‐glucose in the bacterium *Escherichia coli*.

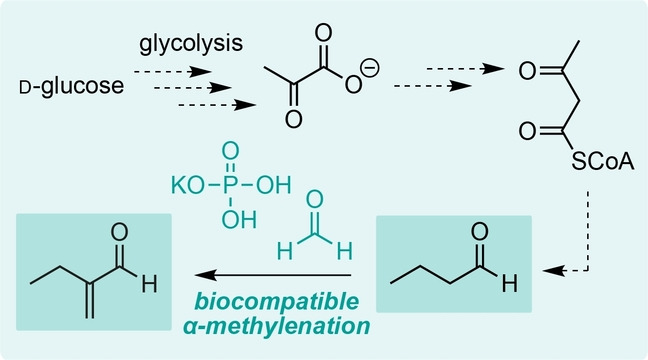

The use of small organic molecules to catalyse chemical reactions (organocatalysis) has transformed our ability to efficiently construct industrially important compounds. Applications of these reactions range from the L‐proline‐catalysed asymmetric aldol reaction in natural product total synthesis, to the functionalisation of biomolecules for cellular labelling and drug delivery in chemical biology.[^1^, ^2^] However, despite the seminal work of Hajos, Parrish, MacMillan, List and others on the L‐proline catalysed aldol reaction, organocatalytic reactions also occur in Nature and are thought to have played an important role in the chemistry of the early Earth (Figure 1A&B).[^3^, ^4^, ^5^] For example, the L‐amino acids Ser, Ala, Phe, Val, Leu, Glu and Pro have been shown to catalyse the formose reaction of glycolaldehyde and formaldehyde under prebiotically relevant conditions.^[6]^ Similarly, Ac−Cys−OH has been shown to catalyse the chemo‐ and regio‐selective peptide ligation of Ac−Gly−CN and L−Ala—suggesting that organocatalysed amide bond formation played a role in the emergence of proteogenic α‐peptides in extant biology.^[7]^ However, despite the abundance of amines in biological systems, and our ability to manipulate the cellular pool of these molecules via genetic engineering, the use of organocatalysed reactions in vivo has received comparatively little attention. This is pertinent as synthetic biology approaches continue to produce compounds of industrial value, and because methods to diversify metabolites accessible via this approach are needed to replace petrochemical feedstocks.[^8^, ^9^, ^10^] To this end, we recently demonstrated the use of tyramine derivatives, and cellular amines from *Corynebacterium glutamicum* as biocompatible organocatalysts for the self‐aldol dimerisation of exogenous butyraldehyde in the presence of *E. coli*.^[11]^ However, the use of biocompatible organocatalysis with engineered metabolic pathways in a living cell remains an outstanding challenge.[^9^, ^12^] Herein we report that L‐proline derivatives and/or cellular phosphate catalyse the non‐enzymatic biocompatible α‐methylenation of butyraldehyde in living *E. coli*. This study represents the first synthesis of 2‐methylenebutanal (2‐MB) from D‐glucose, and the first reported method for the α‐methylenation of cellular aldehydes in a living microorganism.

**Figure 1:**
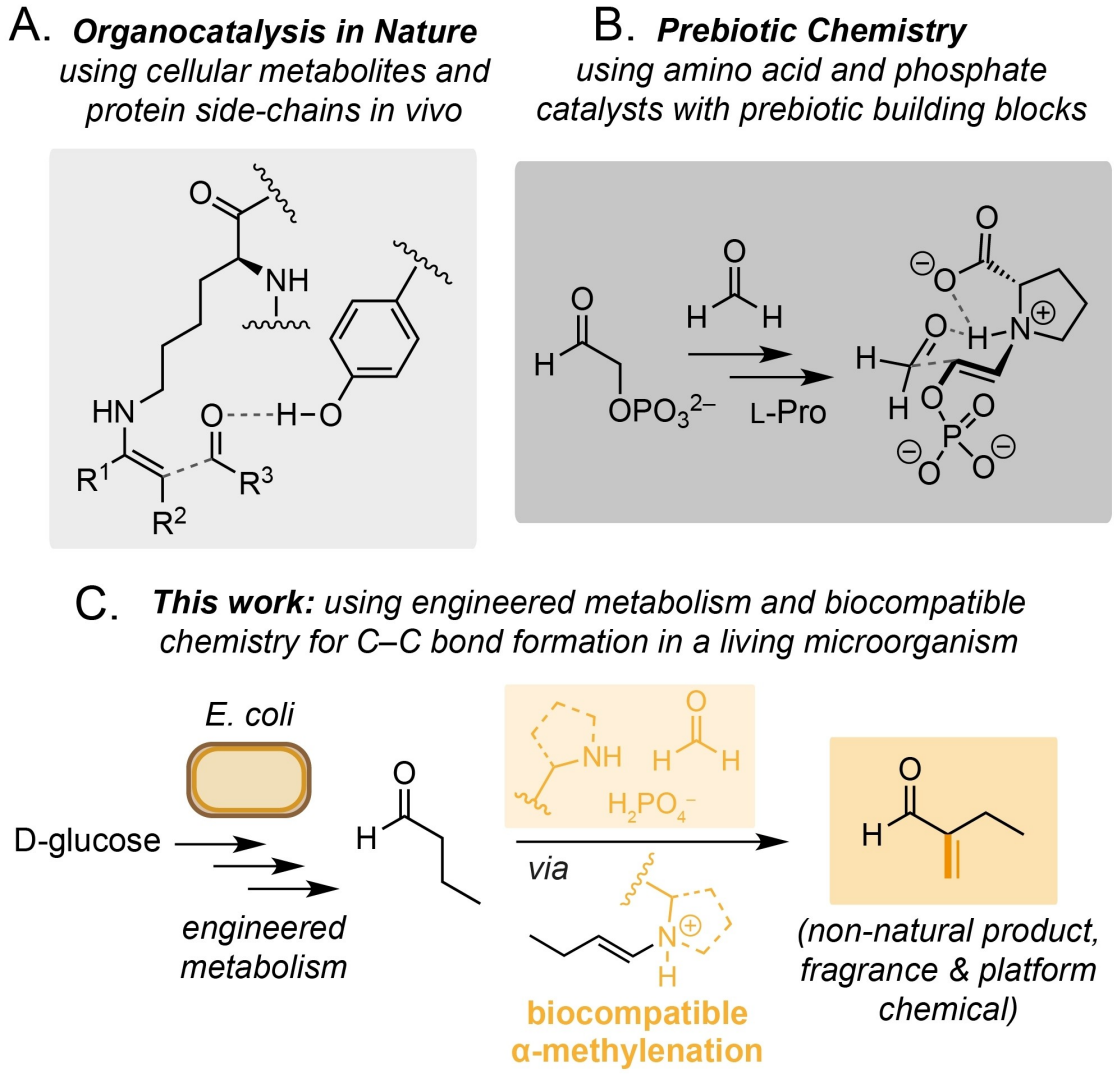
A) Amine organocatalysis in Nature and in synthetic organic chemistry. B) Prebiotic chemistry using amino acid and phosphate organocatalysis. C) Non‐enzymatic α‐methylenation of butyraldehyde generated from D‐glucose via engineered microbial metabolism and biocompatible chemistry.

We began our studies on the α‐methylenation of butyraldehyde for two reasons: (i) butyraldehyde is a known metabolite that can be generated via microbial metabolism, and (ii) the methylenation product, 2‐MB, is a precursor to 2‐methylbutanal (2‐MBA) and 2‐methylbutanol (2‐MBO), which are important industrial chemicals. All four compounds are currently manufactured by chemo‐catalytic hydroformylation via the oxo process from diminishing fossil fuels.^[13]^ To our knowledge, no enzyme is known to catalyse the α‐methylenation or α‐methylation of aldehydes in Nature, and 2‐MB/2‐MBA have never been accessed via microbial fermentation. Furthermore, we have recently reported that *E. coli* possesses a series of putative reductases in its genome with homology to ene‐reductases from *Gluconobacter oxydans* that can catalyze the reduction of α,ß‐unsaturated aldehydes, meaning 2‐MB could potentially be further processed to 2‐MBA via endogenous enzymes when generated in vivo (Table S15).^[11]^ In addition, we were inspired by recent seminal work by Goodwin et al. demonstrating that exogenous α‐ and ß‐amino acids mediate the self‐aldol reaction of aldehydes generated from exogenous alcohols by alcohol oxidases or the bacterium *G. oxydans*.[^14^, ^15^, ^16^] However, the use of non‐enzymatic organocatalytic aldol chemistry with engineered metabolic pathways has yet to be achieved.

We began by examining the effect of butyraldehyde on the growth and viability of *Escherichia coli*. This was necessary as aldehydes are reactive cellular metabolites known to confer toxicity through various mechanisms including DNA interstrand crosslinking, protein crosslinking, depletion of glutathione and thioredoxin, membrane damage, delayed cell division and cell swelling.^[17]^ For this reason we selected *E. coli* KS8, a BW25113(K‐12) derived strain of *E. coli* JCL299 containing 13 knockouts of genes encoding for aldo‐keto reductases, aldehyde dehydrogenases and their associated transcriptional activators (Table S4). Deletion of these genes has been shown to increase aliphatic aldehyde accumulation in vivo.[^18^, ^19^] When we incubated an *E. coli* KS8p3 strain in the presence of exogenous butyraldehyde (25 mM) we observed cell growth and survival, consistent with our previous studies on butyraldehyde dimerisation in the presence of *E. coli* RARE (*R*educed *A*ldehyde *Re*duction).[^11^, ^20^] The viability of *E. coli* KS8p3 was reduced by less than 10‐fold (CFU/mL) in the presence of 25 mM butyraldehyde (Figure 2 and S2). The log‐phase growth of the strain was delayed in the presence of >10 mM butyraldehyde but ultimately reached stationary phase (Figure S3). Although the exact mechanism of butyraldehyde detoxification is currently unclear, this result suggests *E. coli* can mitigate the toxicity of butyraldehyde at high concentrations via endogenous pathways that ultimately support primary metabolic activity and growth. Indeed, the oxidation of butyraldehyde to butyrate is catalysed by a periplasmic aldehyde oxidoreductase (PaoABC) in *E. coli*, which can then enter central fatty acid biosynthesis and degradation pathways as butyryl‐ACP or butyryl‐CoA, respectively.^[21]^ However, when we cultured *E. coli* in the presence of 25 mM butyraldehyde for 24 h and analysed culture supernatant by ^1^H NMR we detected minimal reduction to butanol or oxidation to butyrate (5 % and 0.1 %, respectively). This confirmed that butyraldehyde was biocompatible at this concentration and that endogenous modification of butyraldehyde to unwanted redox products was not a significant competing process under these conditions.

**Figure 2:**
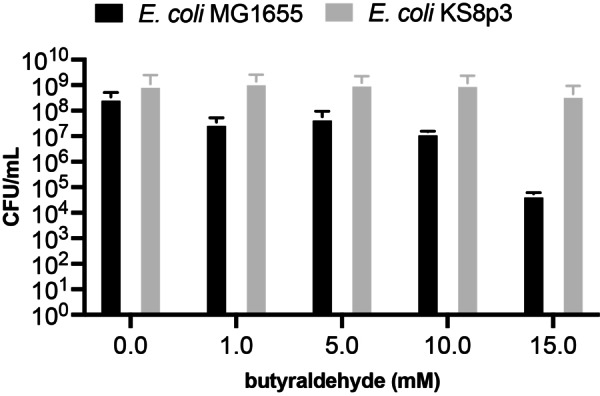
Cell viability experiments in the presence of exogenous butyraldehyde. All data shown is an average of three independent experiments to one standard deviation. The genotype of *E. coli* KS8p3 is shown in Table S1.

Having confirmed the biocompatibility of butyraldehyde to *E. coli* we moved on to examine various organocatalysts for α‐methylenation, focussing on amines that are present in cells, are known to be active under aqueous conditions and/or can be accessed via engineered microbial metabolism. To test this, exogenous amines (Table 1 and S6) were added to ultrapure water alongside butyraldehyde and formaldehyde, incubated at 30 °C at 220 rpm for 24 h before being extracted and analysed by ^1^H NMR. All reactions were screened at pH 7.0 to avoid the self‐aldol dimerization of butyraldehyde. The amine screen included all 20 canonical L‐amino acids, proline congeners and tyramine derivatives (Table 1 and S6–7). Negligible product formation or aldol dimerization to 2‐ethyl‐2‐hexenal was observed in the absence of any additional amine (4 % and <1 % conversion, respectively). The use of many α‐amino acids resulted in low conversions (<20 %) with the exception of the basic amino acids L−Lys and L−Arg (16 % and 21 % 2‐MB, respectively) and the alkyl‐substituted amino acids L−Ile, L−Leu and L−Val (30 %, 35 % and 35 % 2‐MB, respectively). Intriguingly, these amino acids contain either a second amine functional group for organocatalysis or bear a side‐chain with structural resemblance to butyraldehyde. However, L‐proline and *trans*‐hydroxy‐L‐proline were found to be efficient under aqueous conditions, affording 2‐MB in 71 % and 73 % yield, respectively. The biogenic amines tyramine, octopamine and *N*‐methylated analogues were less effective than L‐Pro. The tetrazole‐containing proline analogue reduced the yield of 2‐MB to 63 %, despite being reported as a highly active organocatalyst for the aldol reactions in aqueous media (Table 1, entry 9).^[22]^ The 6‐membered biogenic proline analogue pipecolic acid also reduced the yield of 2‐MB to 11 %, confirming that a pyrrolidine ring is optimal for catalytic activity. We next decided to assess the reactivity of L‐Pro under more bio‐relevant conditions in M9 microbial growth media. To our surprise, despite L‐Pro being active under these conditions (72 % yield), control reactions containing no catalyst produced 2‐MB in 55 % yield. Sequential elimination of the eight components of M9 growth media revealed that the cross‐aldol reactivity was mediated by monobasic potassium phosphate (KH_2_PO_4_), which we hypothesise is less solvated and acts as a Brønsted base under the reaction conditions (Figure 3).^[23]^ This intriguing observation suggests that phosphates could play a dual role in biocompatible reactions by both facilitating cell growth and enabling non‐enzymatic reactions in vivo.

**Table 1:** Screening amine organocatalysts for the α‐methylenation of butyraldehyde.

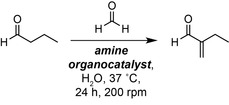			
entry	amine	butyraldehyde (%)	2‐MB (%)
1	–	88	4
2	L‐proline	10	71
3	L‐threonine	61	35
4	L‐valine	56	35
5	L‐isoleucine	56	30
6	L‐arginine	26	21
7	L‐lysine	45	16
8	D,L‐pipecolic acid	95	11
9	(*S*)‐(2‐pyrrolidinyl)‐1H‐tetrazole	14	63
10	*trans*‐4‐hydroxy‐L‐proline	12	73
11	tyramine	41	7
12	octopamine	37	15
13	*N*‐methyltyramine	10	31
14	*N*‐methyloctopamine	11	45

^*a*^ Reaction conditions: Butyraldehyde (25 mM), formaldehyde (25 mM), amine catalyst (25 mM) in ultrapure water. Product concentrations were determined by ^1^H NMR analysis as outlined in Section S1.

**Figure 3:**
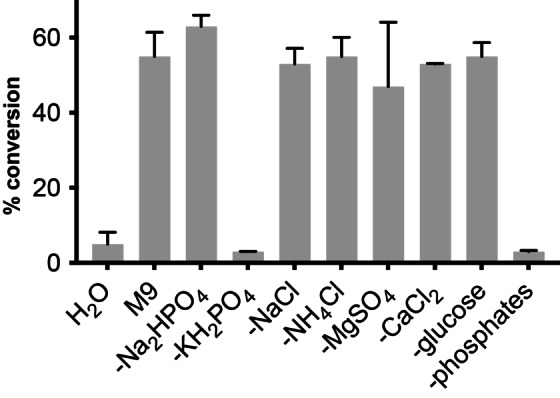
Media component screen in H_2_O, M9 medium or M9 medium minus the specified component. Product concentrations were determined by ^1^H NMR analysis as outlined in Section S1. Data is presented as an average of three independent experiments to one standard deviation.

Having demonstrated that butyraldehyde can undergo an α‐methylenation reaction catalysed by amines and phosphate under aqueous conditions and in M9 microbial growth media, we next moved on to interface this reaction with engineered microbial metabolism. To achieve this, we chose to generate butyraldehyde using an engineered strain of *E. coli* KS8p3 (Figure 4A). This strain generates butyraldehyde from D‐glucose via a redox‐balanced de novo pathway consisting of six heterologous enzymes: a formate dehydrogenase (Fdh) from *Candida boidinii*, acetyl‐CoA acetyltransferase (AtoB) from *E. coli*, 3‐hydroxybutyryl‐CoA dehydrogenase (Hbd) and 3‐hydroxybutyryl‐CoA hydratase (Crt) from *Clostridium acetobutylicum*, *trans*‐enoyl‐CoA reductase (Ter) from *Treponema denticola* and a CoA‐acylating aldehyde dehydrogenase (Aldh) from *Clostridium beijerinckii*. This drives flux from pyruvate generated via glycolysis to acetyl CoA, acetoacetyl CoA, ß‐hydroxyacyl CoA, crotonyl CoA, butyryl CoA and butyraldehyde. The six genes are encoded on pKU48, pRW18 and pRW22 plasmids, and transcription is controlled by fermentative regulatory elements to induce gene expression in the absence of O_2_. Strain KS8 also harbours 13 knock outs (Δ*adhE*, Δ*ldhA*, Δ*frdBC*, Δ*pta*, Δ*yqhD*, Δ*yjgB*, Δ*fucO*, Δ*eutG*, Δ*ybbO*, Δ*adhP*, Δ*gldA*, Δ*yahK*, Δ*yghA*) listed in Table S4 which were shown to decrease the reduction of butyraldehyde to *n*‐butanol in vivo. Altogether, this enables the anaerobic fermentation of D‐glucose to butyraldehyde.[^18^, ^19^] We constructed this strain and confirmed the generation of butyraldehyde from a 24 h fermentation in TB media containing D‐glucose and subsequent incubation at 30 °C at 220 rpm under anaerobic conditions (Figure 4B). To confirm the biocompatibility of the α‐methylenation reaction we first assessed the toxicity of formaldehyde to *E. coli* KS8p3 (Figures S4–5, Table S5). Using cell growth and plate‐count assays, formaldehyde addition resulted in <10‐fold reduction in growth/viability. At 7.5 mM formaldehyde, cell viability (CFU/mL) decreased by 10^6^‐fold. Formaldehyde toxicity was also observed at 5 mM, however cell growth (OD_600_) recovered after 24 h incubation at this concentration, indicating a potential detoxification mechanism in *E. coli* KS8. Indeed, *E. coli* is known to mitigate the toxicity of formaldehyde using glutathione in vivo via expression of the *S*‐(hydroxymethyl)glutathione synthase FrmA. Butyraldehyde was significantly less toxic at 5 mM, resulting in no measurable decrease in cell growth and/or viability (Figures S2–3). Together, this indicated that the α‐methylenation reaction was biocompatible and that formaldehyde detoxification could be a competing side reaction under fermentation conditions.

**Figure 4:**
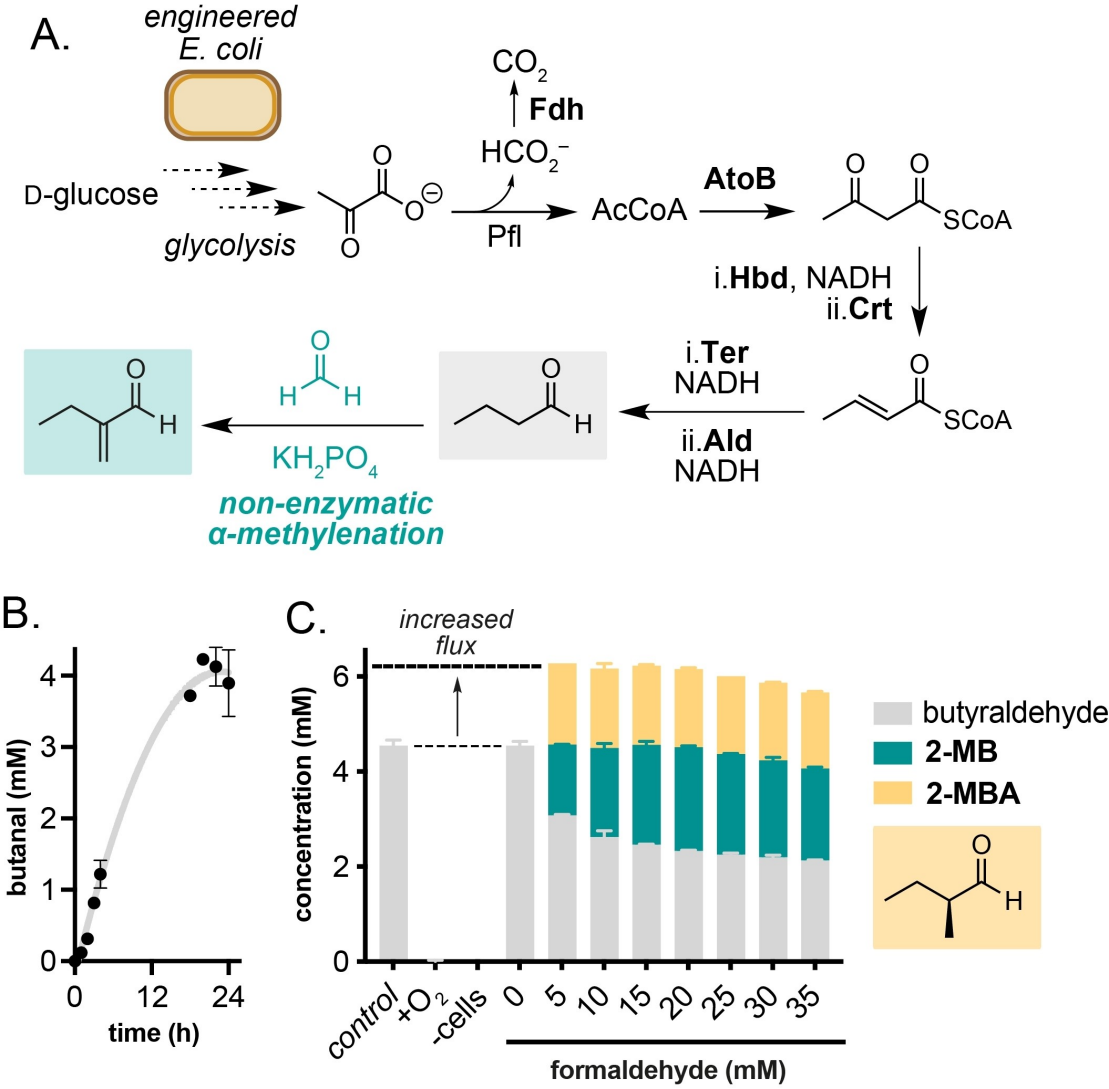
Interfacing butyraldehyde biosynthesis with a biocompatible non‐enzymatic α‐methylenation reaction in engineered *E. coli* KS8p3. A) Engineered metabolic pathway to butyraldehyde from D‐glucose and the biocompatible α‐methylenation step. B) Butyraldehyde production from D‐glucose by *E. coli* KS8p3. C) α‐Methylenation of metabolic butyraldehyde produces methylenation and methylation products and increases metabolic flux. Metabolite concentrations were determined by ^1^H NMR analysis as outlined in Section S1. Data are shown as an average of three independent experiments to one standard deviation.

We began optimising the α‐methylenation reaction with *E. coli* KS8p3 by adding 5 mM formaldehyde to reactions grown to OD_600_=0.5 then sparging with N_2_ to remove O_2_ and induce the butyraldehyde pathway. After 24 h, no butyraldehyde or other products were detected by analysis of the culture supernatant by ^1^H NMR, indicating that formaldehyde was inhibiting the expression and/or activity of the pathway when added at this early timepoint. Replacing formaldehyde with taurolidine enabled the slow release of formaldehyde under these conditions, and indeed led to detectable 2‐MB but in low titres (0.05 mM, 1 % conversion; Table S11). Pleasingly, addition of formaldehyde after 24 h anaerobiosis afforded 2‐MB in 24 % yield in all samples. Analysis of organic extracts of the culture supernatant by ^1^H NMR also identified an additional doublet at δ 9.63 ppm which was identified as 2‐MBA (27 % yield) by comparison to a synthetic standard and attributed to the enzymatic reduction of 2‐MB in vivo by native alkene reductases (Figure S7, Table S15–16). This aligns with our recent observation that unmodified *E. coli* BL21(DE3) cells can reduce the C=C bond of keto‐acrylates via an endogenous mechanism.^[24]^ Together, this result suggested that 2‐MB/2‐MBA could be generated directly from D‐glucose by interfacing engineered metabolism in *E. coli* KS8p3 with a non‐enzymatic biocompatible α‐methylenation reaction catalysed by cellular phosphates. Both 2‐MB and 2‐MBA are small molecules with industrial significance that have not been accessed via engineered metabolic routes and are currently manufactured by multi‐step synthesis from fossil fuels.^[25]^ Furthermore, the total concentration of butyraldehyde, 2‐MB and 2‐MBA in cultures was higher than the concentration of butyraldehyde generated in the absence of the biocompatible reaction, indicating that the non‐enzymatic modification of butyraldehyde in vivo increases flux through butyraldehyde biosynthesis, increasing overall titres; an intriguing and until now unexplored benefit of biocompatible chemistry. 2‐MB and 2‐MBA titres could be further increased to 184 mg/L and 142 mg/L, respectively, by sequential addition of 5 mM formaldehyde over 6 h (Figure 4C).

Having confirmed the biocompatible α‐methylenation of butyraldehyde generated via engineered microbial metabolism, we moved on to explore the reduction of 2‐MB to 2‐methylbutanol (2‐MBO) in *E. coli*. 2‐MBO is an amyl alcohol used in the food and fragrance industries, as well as being a gasoline‐like compound under consideration as a potential biofuel.[^25^, ^26^] De novo metabolic pathways to 2‐MBO have been achieved in *E. coli* from L‐Thr biosynthesis and keto‐3‐methylvalerate, but not from 2‐MB. We reasoned that in addition to C=C bond reduction of 2‐MB to 2‐MBA, endogenous alcohol dehydrogenases and aldo‐keto reductases should enable the in situ biocatalytic reduction of 2‐MBA to 2‐MBO. Overall, this would constitute a novel route to 2‐MBO involving the combined use of engineered metabolism, biocompatible chemistry and whole‐cell biocatalysis. We focussed on the strains *E. coli* KS8p3 and its parental strains *E. coli* KS1p3 and *E. coli* BW25113. *E. coli* KS1p3 has a reduced number of aldehyde reductase genes knockouts (Δ*adhE*, Δ*ldhA*, Δ*frdBC*, Δ*pta*, Δ*yqhD*, Δ*yjgB*) than KS8p3, and *E. coli* BW25113 is the parental K‐12 derived strain. To begin, 2‐MB or 2‐MBA were added to cultures (OD_600_=0.5) of each strain and incubated at 30 °C for 24 h. NMR analysis of reactions after this time showed near‐quantitative reduction of 2‐MBA to 2‐MBO by all strains, and decreased reduction of 2‐MBA to 2‐MBO by *E. coli* KS8p3 at higher substrate concentration (17 % remaining 2‐MBA). We next examined the activity of these strains in the presence of 2‐MB and 2‐MBA under whole‐cell biocatalysis conditions. Promisingly, incubation of 2‐MB in the presence of *E. coli* KS8p3, KS1p3 and BW25113 resuspended to OD_600_=5–50 produced 2‐MBO in all samples (Figure 5). Higher substrate concentrations (≤25 mM) increased mass recovery and yielded 2‐MBO in >99 % yield (30 °C, 24 h, 220 rpm). No 2‐methylenebutan‐1‐ol was detected in any reaction, indicating that the conversion of 2‐MB to 2‐MBO proceeds via initial C=C reduction of the enal followed by reduction of the saturated aldehyde in vivo. Mosher ester analysis of 2‐MBO isolated from whole‐cell reactions confirmed the stereoselective reduction of 2‐MB to the *S*‐enantiomer of 2‐MBA in 77 : 23 e.r (Figure S10 and S11). This is in line with reported C=C reductions of analogous α,ß‐unsaturated substrates by uncharacterised ene‐reductases in *Escherichia sp*.^[23]^ Altogether, this demonstrates a modular approach to the bio‐production of 2‐methyl‐1‐butanol from D‐glucose via the biosynthesis of butyraldehyde, in situ biocompatible α‐methylenation to generate unnatural compounds 2‐methyl‐/2‐methylene‐butanal, followed by stereoselective whole‐cell C=C and C=O reduction by the same microorganism. The reasons why alkene/aldehyde reduction are not engaged under fermentation conditions are unclear, but nevertheless provide conditions whereby the selective access to upstream pathway intermediates can be achieved. Work is currently underway in our laboratory to expand the scope of aldehydes accessible via this strategy, increase the titres of 2‐MB, 2‐MBA and 2‐MBO via genetic and/or chemical in situ product removal (ISPR) methods, and develop new biocompatible reactions to modify butyraldehyde and other metabolic aldehydes.

**Figure 5:**
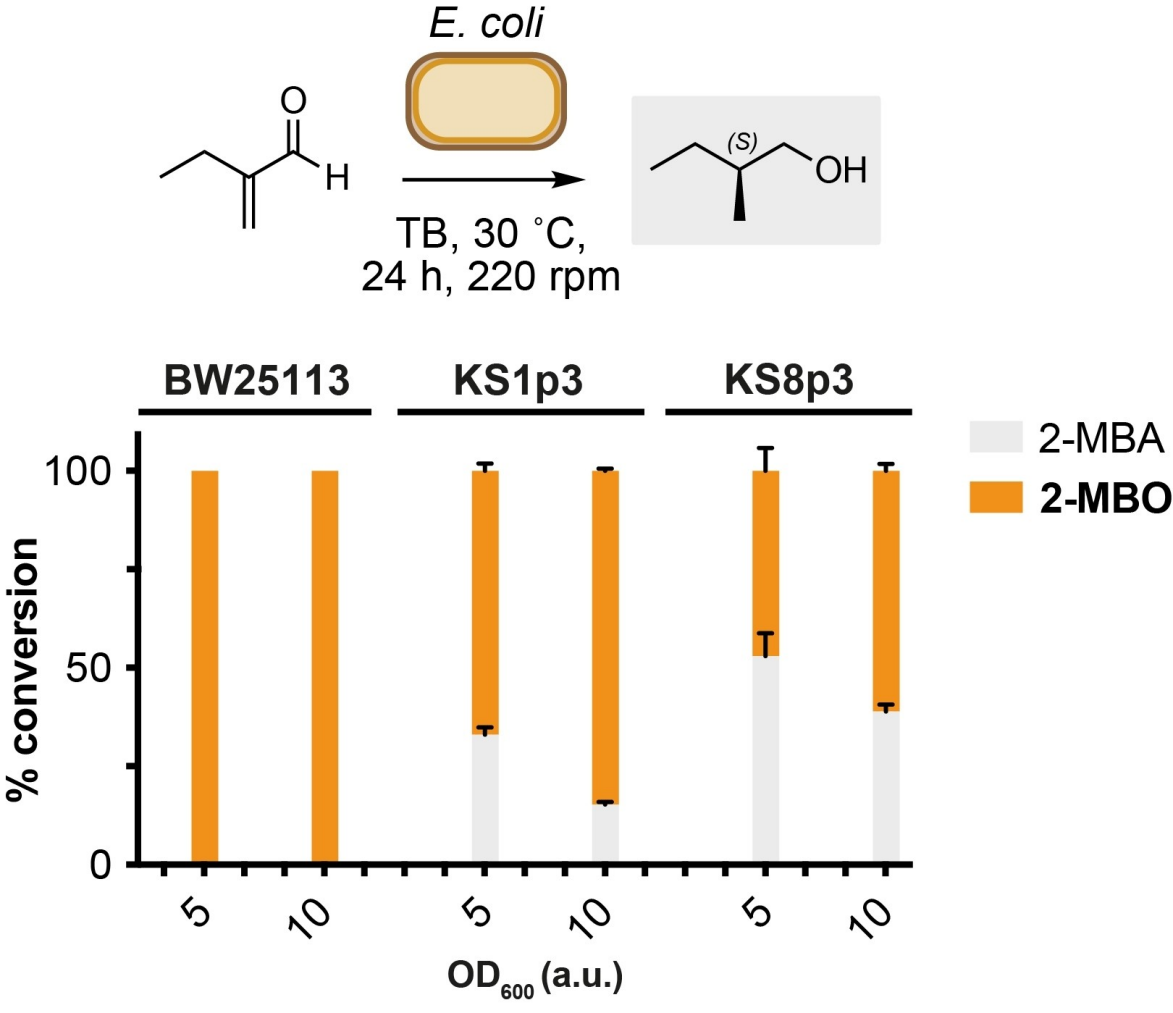
Whole‐cell biotransformation of 2‐MB to 2‐MBA and 2‐MBO by *E. coli* strains. Product concentrations were determined by ^1^H NMR analysis as outlined in S1. Data are shown as an average of three independent experiments to one standard deviation. Substrates were added at 10 mM. (*S*)‐2‐MBO was formed in 77 : 23 e.r. as determined by Mosher ester analysis.

In conclusion, we have achieved the first one‐pot combined bio‐ and chemo‐catalytic synthesis of α‐methylene and α‐methyl aldehydes in living bacteria through the combination of biocompatible chemistry and engineered microbial metabolism. The α‐methylenation of butyraldehyde was shown to be facile under aqueous conditions and catalysed by phosphates present in microbial growth media. The biocompatible reaction could be interfaced with aldehyde biosynthesis from D‐glucose in *Escherichia coli* KS8p3, increasing flux through engineered metabolism and enabling the first bio‐production of these small molecules from sustainable feedstocks. In addition, the reaction was combined with a whole‐cell biotransformation to enable the production of (*S*)‐2‐methyl‐1‐butanol by the same microorganism. Together, these findings demonstrate how engineered cellular metabolism, microbiology and synthetic organic chemistry methods can be combined to create novel routes to important industrial small molecules, and to achieve chemical transformations that are currently inaccessible using enzymatic chemistry alone.

## Author Contributions

The manuscript was written through contributions of all authors. All authors have given approval to the final version of the manuscript.

## Conflict of interest

The authors declare no conflict of interest.

## Supporting information

As a service to our authors and readers, this journal provides supporting information supplied by the authors. Such materials are peer reviewed and may be re‐organized for online delivery, but are not copy‐edited or typeset. Technical support issues arising from supporting information (other than missing files) should be addressed to the authors.

Supporting Information

## Data Availability

The data that support the findings of this study are available in the supplementary material of this article.
