# Supplementary material for: Biocompatible α‐Methylenation of Metabolic Butyraldehyde in Living Bacteria
Source: Angew Chem Int Ed Engl. 2023 Aug 9;62(38):e202306347. doi: 10.1002/anie.202306347 (PMC10952924; doi:10.1002/anie.202306347)
Supplement: Supplementary file 1 — Supporting Information [file ANIE-62-0-s001.pdf]

Angewandte Chemie
Eine Zeitschrift der Gesellschaft Deutscher Chemiker

Supporting Information

Biocompatible α-Methylenation of Metabolic Butyraldehyde in Living Bacteria

*J. A. Dennis, N. W. Johnson, T. W. Thorpe, S. Wallace**

Supplementary Information

Biocompatible α-Methylenation of Metabolic Butyraldehyde in Living Bacteria

Jonathan A. Dennis,[1,2] Nick W. Johnson,[1] Thomas W. Thorpe[1] and Stephen Wallace[1*]

[1]Institute of Quantitative Biology, Biochemistry and Biotechnology, School of Biological Sciences, University of Edinburgh, Edinburgh, EH9 3FF, UK

[2]EaStCHEM School of Chemistry, University of Edinburgh, Edinburgh, EH9 3FJ, UK

*correspondence to: stephen.wallace@ed.ac.uk

S1 General methods 3
S1.2 Media, strains and culturing conditions 4
S1.3 Strains and plasmids 6
S2 Experimental methods 8
S2.1 Butyraldehyde and formaldehyde toxicity screen 8
S2.2 α-Methylenation catalyst screen 8
S2.3 M9 medium composition 9
S2.4 Butyraldehyde biosynthesis procedure 9
S2.5 Biocompatible α-methylenation procedure 9
S2.6 Biocompatible formal α-methylation 10
S2.7 Whole-cell biotransformation of 2-methylenebutanal and 2-methylbutanal 11
S2.8 Esterification of bio-derived 2-MBO with Mosher's acid 11
S3 Supplementary data 12
S3.1 Butyraldehyde and formaldehyde toxicity screen 12
S3.2 α-Methylenation in vitro catalyst screening data 14
S3.3 M9 media composition screen 16
S3.4 Butyraldehyde production time-course 17
S3.5 Biocompatible α-methylenation of butyraldehyde 18
S3.6 Biocompatible formal α-methylation of butyraldehyde 20
S3.7 Whole-cell bioreduction of 2-MB by *E. coli* strains 21
S3.8 Mosher ester analysis of bio-derived 2-MBO 25
S4 References 26

S1 General methods

S1.1 General materials and methods

NMR: ^{1}H, ^{13}C and ^{19}F nuclear magnetic resonance (NMR) spectra were acquired using a Bruker AVA 500 or PRO 500 (500 MHz) NMR spectrometer at 20 °C. Proton chemical shifts are expressed in parts per million (ppm, δ scale) and are referenced to residual protium in the NMR solvent ($CDCl_3$, δ 7.26 ppm). Coupling constants, J, are measured to the nearest 0.1 Hz and are presented as observed. Data is represented as: chemical shift, integration, multiplicity (s = singlet, d = doublet, t = triplet, q = quartet, dd = doublet of doublet, m = multiplet and/or multiple resonances), coupling constant (J) in Hertz. For all quantitative measurements by ^{1}H-NMR spectroscopy 1,3,5-trimethoxybenzene (TMB, 2 mM) was used as an internal standard.

All chemicals and solvents were purchased from commercial suppliers and were used without further purification. All catalysts used in the initial screen were purchased from Sigma Aldrich or Fluorochem UK. All water used experimentally was purified with a Suez Select purification system (18 mΩ cm^{-1}, 0.2 μm filter). All NMR solvents were purchased from Sigma Aldrich. Optical densities of *E. coli* cultures were determined using a DeNovix DS-11 UV-Vis spectrophotometer by measuring absorbance at 600 nm.

Metabolites were extracted and analysed by ^{1}H NMR as follows: Sealed hungate tubes containing spent reaction samples were cooled in a freezer to condense the headspace (-20 °C, 15 min) and then unsealed. An aliquot of the reaction (0.5 mL) was added to a 2 mL microcentrifuge tube containing an equal volume of brine. NMR solvent (0.33 mL $CDCl_3$ containing 2 mM TMB) was added to the microcentrifuge tube which was then vortexed for 10 minutes and centrifugated (12,000 × g, 5 min). The organic subnatant was removed using a Pasteur pipette and transferred to a 1.5 mL microcentrifuge tube. Twice more, NMR solvent (0.33 mL containing 2 mM TMB) was added to the aliquot, vortexed and centrifugated again to obtain a total of approximately 1 mL of extract in NMR solvent containing TMB as an internal standard. Anhydrous Na_2SO_4 was added to dry the samples. The full volumes of deuterated NMR solvent were then analysed by ^{1}H NMR as outlined above.

S1.2 Media, strains and culturing conditions

Lysogeny broth (LB) was prepared according to the following procedure: bacto-tryptone (10 g/L), yeast extract (5 g/L) and NaCl (10 g/L) were dissolved in ultrapure H_2O. LB was autoclaved at 121 °C for 20 min, cooled and stored at room temperature. LB agar was made using the same recipe but with the addition of agar (15 g/L). SOC media was prepared according to the following procedure: bacto-tryptone (20 g/L), yeast extract (5 g/L) and NaCl (0.5 g/L) were dissolved in ultrapure H_2O. KCl was added to a final concentration of 2.5 mM. The mixture was autoclaved at 121 °C for 20 min and cooled before adding glucose (20 mM) and $MgCl_2$ (10 mM). SOC media was then stored at room temperature. Terrific Broth (TB) medium was prepared according to the following procedure: bacto-tryptone (20 g/L), yeast extract (24 g/L) and glycerol (4 mL/L) were dissolved in ultrapure H_2O. TB was autoclaved at 121 °C for 20 min. Filter sterilised phosphate buffers were added to the cooled solution to a final concentration of 0.17 M KH_2PO_4 and 0.72M K_2HPO_4 after which the TB was stored at room temperature.

M9 minimal medium was made by first preparing individual components as stock solutions according to the following procedure: Concentrated (10×) M9 salts (500 mL) were prepared by dissolving either Na_2HPO_4 (75.2 g/L), KH_2PO_4 (30 g/L), NaCl (5 g/L) or NH_4Cl (5 g/L) in ultrapure water (500 mL) in Duran bottles, which were then autoclaved. Glucose (20 % w/v) was dissolved in water and autoclaved. Stock solutions of $MgSO_4$ (1M) and $CaCl_2$ (1M) were prepared by dissolving the salts in ultrapure water, and filter sterilising. Stock solutions of biotin (1 mg/mL) and thiamine monohydrochloride (1 mg/mL) were prepared by dissolving the respective compounds in ultrapure water and filter sterilising. Trace elements stock solution (100×, 500 mL) was prepared by dissolving EDTA (2.5 g) in ultrapure water (400 mL). The pH was adjusted to 7.5 using NaOH solution (2 M). The following compounds were then added to the EDTA solution: $FeCl_3$ (249 mg), $ZnCl_2$ (84 mg), $CuCl_2 \bullet 2H_2O$ (382.5 µL of 0.1 M solution, $CoCl_2 \bullet 6H_2O$ (105 µL of 0.2 M solution), H_3BO_3 (800 µL of 0.1 M) and $MnCl_2 \bullet 4H_2O$ (4.1 µL of 1 M stock). The trace elements stock solution was then filter sterilised. Stock solutions were added to ultrapure water (~500 mL) as follows: M9 salts (4 × 100 mL of 10× stocks (400 mL total)), glucose (20 mL), $MgSO_4$ (1 mL), $CaCl_2$ (0.3 mL), biotin (1 mL), thiamine (1 mL) and trace elements solution (10 mL of 100×). Ultrapure water was added to a total volume of 1000 mL.

All other reagents used in culturing experiments were filter sterilised using a 0.22 µm filter (Millex). All chemically competent cells were prepared via treatment with calcium chloride. For use as a culture inoculum, chemically competent *E. coli* KS8 (Tet^r) was prepared and transformed with plasmids pRW18 (Cam^r), pRW22 (Kan^r) and pKU48 (Amp^r) via heat-shock at 42 °C for 45 s. Cells were recovered in 1 mL of SOC media for 1 h at 37 °C. Transformants were selected by plating on LB agar containing appropriate antibiotics and incubated overnight at 37 °C. A single colony was picked and

grown overnight in 10 mL of LB containing appropriate antibiotics. The resulting overnight culture (0.5 mL) was added to 0.5 mL of 1:1 v/v water:glycerol solution, frozen in liquid nitrogen, stored at –80 °C and used as required. For microbiological experiments, antibiotics were used at the following concentrations: ampicillin (Amp), 100 μg/mL; chloramphenicol (Cam), 50 μg/mL; kanamycin (Kan), 50 μg/mL; tetracycline (Tet), 15 μg/mL. Antibiotics were prepared as stock solutions (500−1000 times concentrated) and filter sterilized before use.

E. coli KS8 cells and plasmid pKU48 were obtained from Prof. Ethan Lan (National Chiao Tung University, Taiwan). Plasmids pRW18 and pRW22 were obtained from Prof. Claire Shen (National Tsing Hua University, Taiwan). Transformation of KS8 cells, as outlined below, with plasmids pKU48, pRW18 and pRW22 resulted in strain *E. coli* KS8p3. All cells were stored as 1:1 v/v LB:glycerol stocks at −80 °C and used as required.

Engineered *E. coli* KS8 was prepared from parent strain *E. coli* JCL299 (itself derived from *E. coli* BW5113) by sequentially deleting aldehyde reductase genes as outlined by Ku and co-workers.[1] All gene knock-outs were performed using P1 transduction with the Keio collection as donor strains. The butyraldehyde production pathway is under the control of fermentative regulatory elements, which allow for induction of the pathway upon sparging cultures with nitrogen to remove oxygen.

S1.3 Strains and plasmids

Table S1: *E. coli* strains used in this study

Strain	Plasmid(s)	Genotype	Source
E. coli BW25113	pET_22b (empty vector)	*rrnB* $_{T14}$ Δ*lacZ* $_{WJ16}$ hsdR514 Δ*araBAD* $_{AH33}$ Δ*rhaBAD* $_{LD78}$	Addgene
E. coli KS1	-	BW25113/F' [traD36 proAB $^{+}$ lacIqZΔM15 (Tet^{r})] Δ*adhE* Δ*ldhA* Δ*frdBC* Δ*pta* Δ*yqhD* Δ*yjgB*	[1]
E. coli KS8	-	KS1 Δ*fucO* Δ*eutG* Δ*ybbO* Δ*adhP* Δ*gldA* Δ*yahK* Δ*yghA*	[1]
E. coli KS1p3	pRW18 pRW22 pKU48	BW25113/F' [traD36 proAB $^{+}$ lacIqZΔM15 (Tet^{r})] Δ*adhE* Δ*ldhA* Δ*frdBC* Δ*pta* Δ*yqhD* Δ*yjgB*	[1]
E. coli KS8p3	pRW18 pRW22 pKU48	KS1p3 Δ*fucO* Δ*eutG* Δ*ybbO* Δ*adhP* Δ*gldA* Δ*yahK* Δ*yghA*	[1]

Table S2: Plasmids used in this study

Plasmid(s)	Marker	Genotype	Source
pET-22b(+) (empty vector)	Amp^{r}		Prof. Chris French (University of Edinburgh)
pRW18	Cam^{r}	P_{adhE}::*fdh* (*Candida boidinii*); pSC101 ori	[2]
pRW22	Kan^{r}	P_{adhE}::*ter* (*Treponema denticola*); Cola ori	[2]
pKU48	Amp^{r}	P_{ack}::*atoB* (*Escherichia coli*), *aldh* (*Clostridium beijerinckii*), *crt* (*Clostridium acetobutylicum*), *hbd* (*Clostridium acetobutylicum*); ColE1 ori	[1]

Table S3: Butyraldehyde pathway enzymes present on plasmids pRW18, pRW22 and pKU48

Entry	Enzyme	Enzyme Name	Native Organism
1	Fdh	formate dehydrogenase	*Candida boidinii*
2	Ter	trans-enoyl-CoA reductase	*Treponema denticola*
3	AtoB	acetyl-CoA acetyltransferase	*Escherichia coli*
4	Aldh	CoA-acylating aldehyde dehydrogenase	*Clostridium beijerinckii*
5	Crt	3-hydroxybutyryl-CoA hydratase	*Clostridium acetobutylicum*
6	Hbd	3-hydroxybutyryl-CoA dehydrogenase	*Clostridium acetobutylicum*

Table S4: Genes knocked out in *E. coli* KS8p3

Entry	Gene	Enzyme Name	Native (Oxidised) Substrate(s)
1	*adhE*	Acetaldehyde dehydrogenase	Acetaldehyde
2	*ldhA*	Lactate dehydrogenase A	Pyruvate
3	*frdBC*	Fumarate reductase	Fumarate, quinone
4	*pta*	Phosphate acetyltransferase	Acetate, CoA
5	*yqhD*	4-hydroxy-2-ketovalerate aldolase / acetaldehyde dehydrogenase	4-hydroxy-2-ketovalerate, acetaldehyde
6	*yjgB*	Uncharacterised oxidoreductase	Aldehydes
7	*fucO*	L-fucose dehydrogenase	L-fucono-1,5-lactone
8	*eutG*	Ethanolamine-phosphate cytidylyltransferase	Ethanolamine phosphate
9	*ybbO*	Uncharacterised aldehyde reductase	Aldehydes
10	*adhP*	Alcohol dehydrogenase P	Acetaldehyde
11	*gldA*	Glycerol dehydrogenase	Glyceraldehyde
12	*yahK*	NAD(P)H-dependent aldehyde reductase	Short-chain aldehydes
13	*yghA*	NADPH-dependent aldehyde reductase	Short-chain aldehydes

S2 Experimental methods

S2.1 Butyraldehyde and formaldehyde toxicity screen

A 250 mL culture of *E. coli* KS8p3 in LB medium was grown to OD_{600} = 0.5−0.6 as outlined in S1.2, and 7.5 mL of the resulting culture was added to sterile hungate tubes. Using a gas tight syringe, a range of butyraldehyde concentrations (1−25 mM) or formaldehyde (0.5−10 mM) were added to the cultures. The tubes were then sealed using butyl rubber septa and incubated horizontally at 30 °C, 220 rpm. Cell density measurements were taken at regular intervals for the first 4 hours by measuring the OD_{600} of the culture tubes. After 24 h, 100 μL of each reaction mixture was removed and added to 900 μL of ultrapure water. These aliquots were subjected to serial 10-fold dilutions (10^1–10^8). Aliquots (100 μL) of each dilution were plated onto individual LB agar plates containing ampicillin, kanamycin, chloramphenicol and tetracycline. The plates were incubated at 30 °C overnight and the number of colonies from these plates were used to calculate the number of colony-forming units (CFUs) in each mL of culture.

Minimum inhibitory concentration (MIC) experiments were performed in 96-well plates in triplicate using butyraldehyde (0–250 mM) or formaldehyde (0–10 mM). LB medium containing appropriate antibiotics was added to each well to a total volume of 390 μL, and then 10 μL of a freshly grown overnight culture of *E. coli* BW25113, *E. coli* KS8 or *E. coli* KS8p3 was added. The plate was incubated overnight (37 °C, 50 rpm), and the optical density at 600 nm (OD_{600}) of each well was determined using a plate reader. With comparison to the negative control without aldehyde, and sterile LB medium, the minimum concentration at which each strain did not grow was determined.

S2.2 α-Methylenation catalyst screen

All catalyst screening reactions were carried out in triplicate using autoclaved 15 mL glass hungate tubes with butyl rubber septa and screw caps. Tubes contained 7.5 mL reaction volume and 7.5 mL headspace. The initial α-methylenation catalyst screen was performed using ultrapure water at pH 7.4 as the reaction medium. Various amines (25 mM) were weighed directly into sterile hungate tubes, followed by ultrapure water (7.5 mL). The tubes were vortexed gently to dissolve the amine compound, after which butyraldehyde (25 mM, 17 μL) was added using a gas-tight syringe. Formaldehyde (25 mM) was then added as a 4% w/v solution in water using a micropipette. The hungate tubes were sealed and then incubated horizontally (24 h, 30 °C, 200 rpm). Aliquots of each reaction were then extracted and analysed by ^{1}H NMR as outlined in S1.1.

S2.3 M9 medium composition

Variants of M9 medium were prepared by individually omitting stock solutions from the recipe outlined in S1.2 and replacing the stock solution with an equal volume of sterile ultrapure water. Either Na_2HPO_4, KH_2PO_4, NaCl, NH_4Cl, $MgSO_4$, $CaCl_2$, glucose or phosphates were omitted to investigate the effects of each component on the yield of the α-methylenation reaction, as outlined in S2.2, in the absence of catalyst. Trace elements stock solution was omitted from all in vitro experiments with M9 media due to the low concentration of each component.

S2.4 Butyraldehyde biosynthesis procedure

E. coli cultures were prepared by inoculating 10 mL of LB (containing Amp, Cam, Kan and Tet) with a –80 °C LB:glycerol stock of *E. coli* KS8p3 and incubating cultures at 30 °C (220 rpm) for 18 h. The saturated overnight culture (5 mL) was then inoculated into TB medium + 2% w/v D-glucose (250 mL in 500 mL Erlenmeyer flask) containing Amp, Cam, Kan and Tet, and grown aerobically at 220 rpm until the culture reached OD_{600} = 0.5−0.6 (ca. 2.5−3 h). The cultures were then transferred to hungate tubes (7.5 mL culture in 15 mL hungate tube) and sealed with butyl rubber septa and screw-caps. The reactions were sparged with nitrogen gas for 10 min using a 21-gauge, 4.25-inch needle as the inlet and a 25-gauge, 0.63-inch needle as the outlet. The cultures were incubated at 30 °C (200 rpm) for 24 h. Aliquots were then extracted and analysed by ^{1}H NMR as outlined in S1.1.

S2.5 Biocompatible α-methylenation procedure

Cultures of *E. coli* KS8p3 were grown aerobically as outlined above in S2.4. L-Proline (5 mM) was added to the shake flask containing the culture and then gently swirled to dissolve the catalyst, before transferring 7.5 mL to hungate tubes. The butyraldehyde production pathway was induced by sealing and sparging the tubes with N_2 gas to create an anaerobic growth environment. After 24 hours incubation at 30 °C, 220 rpm, the hungate tubes were placed in a freezer (–20°C) for 15 min to condense the culture headspace. Formaldehyde (5 mM of 4% (w/v) in H_2O, 28.2 μL) was added to the tubes using a micropipette by quickly unsealing and resealing the tubes. The hungate tubes were then placed back in the incubator for 1 h, after which formaldehyde was added again. This process was repeated until a total of 35 mM formaldehyde had been added. After 24 h since the initial addition of formaldehyde, aliquots were taken for analysis by ^{1}H NMR as outlined in S1.1.

S2.6 Biocompatible formal α-methylation

Cultures of *E. coli* KS8p3 were grown aerobically as outlined in S2.4. L-Proline (5 mM) was added to the culture and then gently swirled to dissolve the organocatalyst. The culture (5 mL) was transferred to hungate tubes containing the appropriate amount of taurolidine. The butyraldehyde production pathway was induced by sealing and sparging the tubes with N_2 gas to create an anaerobic growth environment. After 24 hours incubation, the hungate tubes were cooled (15 min, –20 °C) and aliquots were extracted and analysed by ^{1}H NMR as outlined in S1.

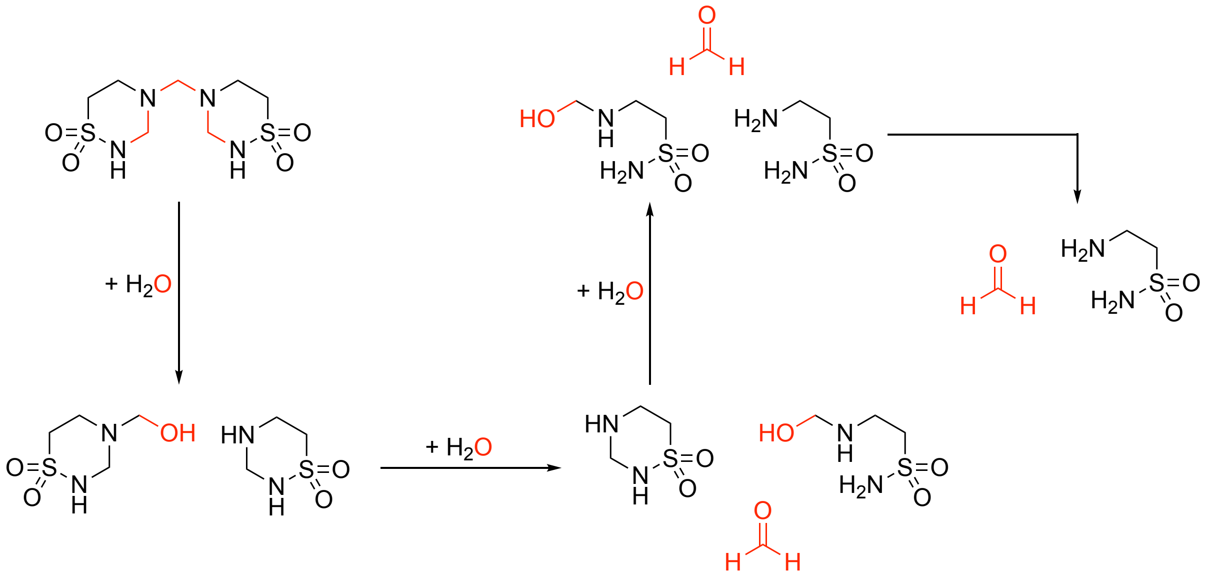

Figure S1: Structure of taurolidine and mechanism of formaldehyde release

S2.7 Whole-cell biotransformation of 2-methylenebutanal and 2-methylbutanal

Cultures of *E. coli* BW25113, *E. coli* KS1p3 or *E. coli* KS8p3 in TB medium supplemented with 2% w/v glucose (1 L) and appropriate antibiotics were prepared by inoculating with a fresh overnight culture as outlined in S1.2. Cells were grown to a cell density of either OD_{600} = 0.5 or 5 (approximately at 3 h or 18 h incubation respectively). The culture was centrifugated (10,000 × g, 10 min, 4 °C) in order to collect the cells and the supernatant was discarded. The cell pellet was resuspended in fresh medium (TB + 2% glucose) to the desired cell density (OD_{600} = 0.5–50). Aliquots of the culture (7.5 mL) were added to sterile 15 mL hungate tubes, followed by varying concentrations of either 2-MB or 2-MBA using a glass gas-tight syringe. The sealed tubes were then incubated horizontally for 24 h (30 °C, 220 rpm). Aliquots of the culture were sampled for ^{1}H NMR analysis as outlined in S1.1. Careful regard was given at higher cell densities not to disturb the densely packed interfacial pellet comprised of cell debris and membranes. This was achieved by pushing the pellet aside with the tip of a glass Pasteur pipette, extracting as much of the organic subnatant as possible, centrifuging the sample and then extracting the remaining organic subnatant.

S2.8 Esterification of bio-derived 2-MBO with Mosher's acid

Cultures of *E. coli* KS8p3 were grown and resuspended to a cell density of OD_{600} = 5 as outlined in S2.7. To a sealed 500 mL shake flask containing 250 mL of culture, 2-MB (2 mM, 0.5 mmol) was added. After 24 h incubation, the culture was extracted into ice cold diethyl ether (2 × 250 mL) and concentrated in vacuo to afford a clear brown oil. The crude extract was derivatised with (R)-α-methoxy-α-trifluoromethylphenylacetic acid ((R)-MTPA) using an adapted procedure to determine the enantiomeric excess of bio-derived 2-methyl-1-butanol compared to commercial standards of rac- and (S)-2-MBO derivatised under analogous conditions.[3]

Synthetic procedure: To a solution of crude extracted 2-methyl butanol, N,N-dimethylaminopyridine (3 mg, 26 μmol, 0.05 equiv.), (R)-MTPA (107.7 mg, 0.46 mmol, 0.90 equiv.) in 9 mL of CH_2Cl_2 was added dropwise to a solution of dicyclohexylcarbodiimide (106 mg, 0.511 mmol, 1.00 equiv.) in 9 mL of CH_2Cl_2. The resulting mixture was stirred for 18 h at room temperature before being diluted in cyclohexane and filtered. The filtrate was washed with HCl (15 mL, 1 M) $NaHCO_3$ (15 mL, sat.) and brine (15 mL, sat.) before being dried with Na_2SO_4, filtered and evaporated. The resulting crude material was purified by silica column chromatography (98:02 ethyl acetate:cyclohexane) to afford the (R)-MTPA ester ((S)-54% e.e. by ^{1}H NMR) as an oil. NMR data was in good agreement with literature data.[3]

S3 Supplementary data

S3.1 Butyraldehyde and formaldehyde toxicity screen

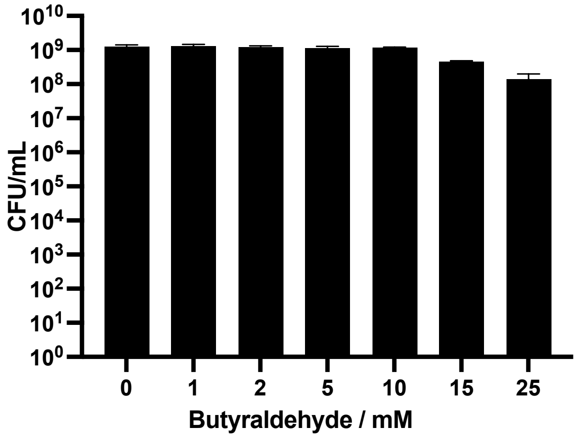

Figure S2: CFU assay comparing the effects of butyraldehyde (1−25 mM) on the viability of *E. coli* KS8p3. Error bars represent the standard deviation of experiments performed in triplicate as outlined above.

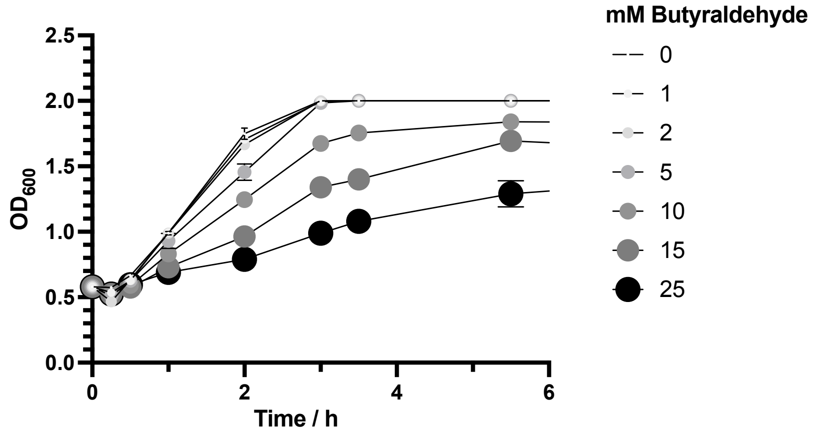

Figure S3: Cell density assay comparing the effects of butyraldehyde (1−25 mM) on the viability of *E. coli* KS8p3. Error bars represent the standard deviation of experiments performed in triplicate as outlined above.

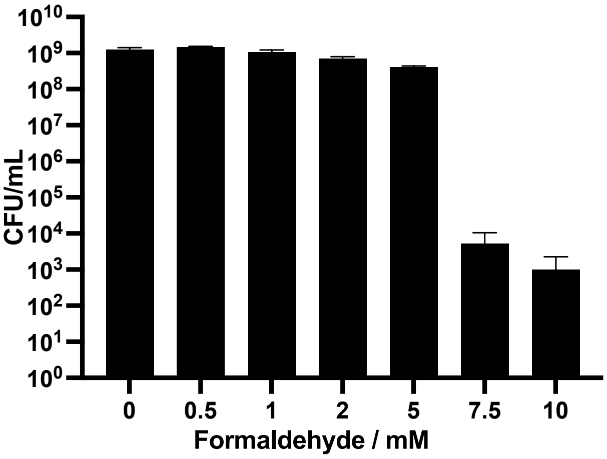

Figure S4: CFU assay comparing the effects of formaldehyde (0.5−10 mM) on the viability of *E. coli* KS8p3. Error bars represent the standard deviation of experiments performed in triplicate as outlined above.

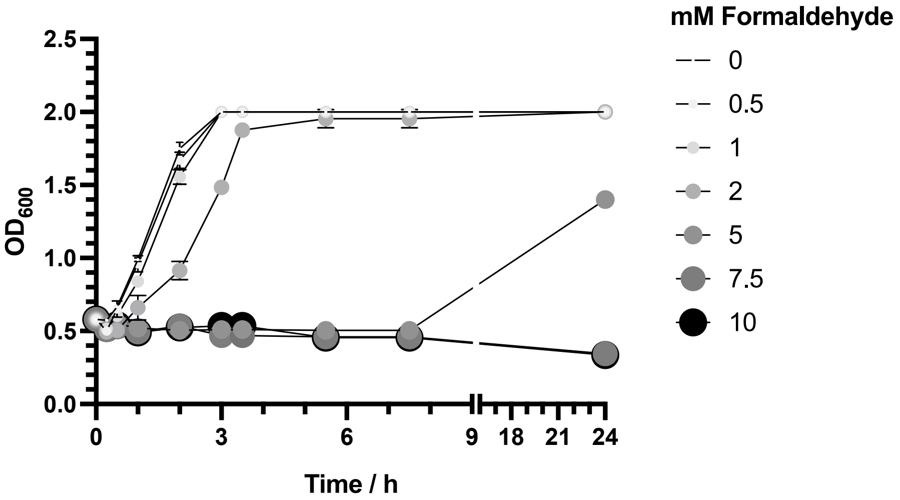

Figure S5: Cell density assay comparing the effects of formaldehyde (0.5−10 mM) on the viability of *E. coli* KS8p3. Error bars represent the standard deviation of experiments performed in triplicate as outlined above.

Table S5: Minimum inhibitory concentration of butyraldehyde and formaldehyde.

***E. coli* Strain**	**Minimum Inhibitory Concentration (mM)**	
	**Formaldehyde**	**Butyraldehyde**
BW25113	9	250
KS8	7	250
KS8p3	6	200

S3.2 α-Methylenation in vitro catalyst screening data

Table S6: Catalyst screen, with ±SD representing the standard deviation of experiments performed in triplicate as outlined in S2.2. Yield % is calculated from concentrations determined by ^{1}H NMR analysis of the crude reaction extract.

Catalyst	Butyraldehyde		2-MB		2-ethyl-2-hexenal	
	%	SD	Yield %	SD	Yield %	SD
None	87.1	1.0	3.8	0	8.6	0
Casamino Acids	54.75	5.4	26.15	3.7	10.6	0
L-Arginine	25.2	5.1	20.85	3.7	16.8	2.0
L-Lysine	44.65	0.6	15.85	0.6	15.35	2.8
L-Proline	9.35	0.4	70.55	5.4	9.1	0.77
L-Alanine	80.9	5.8	17.3	0	12	0.7
Glycine	69.1	4.1	9.35	0.4	12.45	1.3
L-Isoleucine	55.45	0.4	29.75	2.8	12	0.7
L-Leucine	67.2	1.4	23.05	1.3	13.45	1.3
L-Valine	55.2	2.0	34.55	0.6	12.45	1.3
L-Phenylalanine	55.2	1.4	27.15	0.4	13.9	0.7
L-Tryptophan	77.25	1.3	15.15	0.4	12.45	1.3
L-Tyrosine	90.25	0.6	9.85	0.4	11.5	0
L-Aspartic Acid	98.4	2.0	6.7	0	11.5	0
L-Glutamic Acid	96.25	0.4	9.6	0	11.5	0
L-Histidine	96	0.7	5.3	0.7	12.5	0
L-Serine	58.3	2.4	24.45	1.3	13.4	0
L-Threonine	60.75	7.1	34.8	5.8	13.9	0.7
L-Cysteine	61.7	2.4	7.45	0.4	11.05	0.6
L-Methionine	57.85	0.4	24.5	0.7	12.5	0
L-Asparagine	81.6	2.7	9.1	0.7	12	0.7
L-Glutamine	62.15	0.4	24.75	0.4	13.9	0.7
Trans-4-Hydroxy-L-proline	11.75	0.4	72.45	5.4	9.6	0
S-Proline Tetrazole	13.2	1.0	62.15	7.1	9.6	0
L-Proline-β-Naphthylamide	64.1	1.7	7.7	0	8.6	0
L-Pipecolic Acid	94.3	8.5	10.1	0.7	9.6	0

Catalyst	Butyraldehyde		2-MB		2-ethyl-2-hexenal	
	**%**	**SD**	**Yield %**	**SD**	**Yield %**	**SD**
Histamine	77	1.7	6.7	0.7	12.45	1.3
Agmatine	83.25	3.7	7.95	0.4	14.4	1.4
6-aminohexanoic acid	76.8	1.4	7.2	0	10.1	0.7
β-alanine	62.85	1.3	10.1	0	14.4	0
Tyramine	40.05	0.4	6.2	0	16.35	1.3
Octopamine	36.95	0.6	14.15	0.4	18.25	2.7
N-methyltyramine	9.6	0	30.45	0.4	9.6	0
N-methyloctopamine	10.05	1.3	44.65	2.8	9.6	0
Hordenine	80.65	4.7	7.95	0.4	11.5	0

Table S7: Structures of biogenic amines screened for catalytic activity

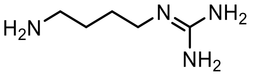	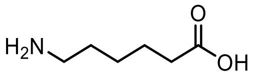	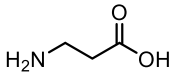	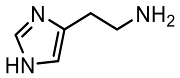
agmatine	6-aminohexanoic acid	ß-alanine	histamine
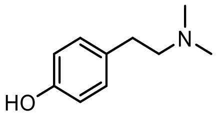	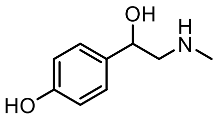	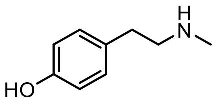	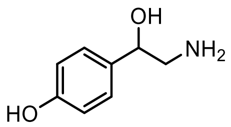
hordenine	N-methyl octopamine	N-methyltyramine	octopamine
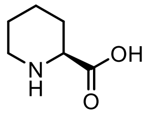	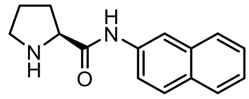	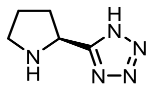	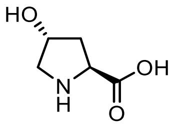
L-pipecolic acid	L-proline-ß-naphthylamide	(S)-5-(pyrrolidine-2-yl)tetrazole	trans-4-hydroxy-L-proline
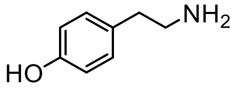			
tyramine			

S3.3 M9 media composition screen

Table S8: Results of M9 media composition screen performed as outlined in S2.3. Yield % is calculated from concentrations determined by ^1H NMR analysis of the crude reaction extract.

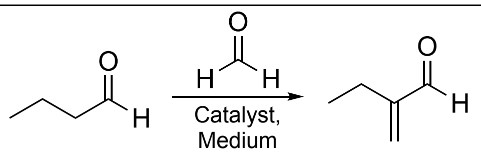

Medium	Proline / mM	% Butyraldehyde	% 2-MB
H_2O	0	81 ± 0.2	5 ± 3.2
	25	9.4 ± 0.4	71 ± 5.4
M9	0	26 ± 4.0	55 ± 6.4
	25	11 ± 0.2	47 ± 1.4
M9 minus Na_2HPO_4	0	8 ± 0.1	63 ± 3.0
	25	19 ± 2.0	43 ± 1.5
M9 minus KH_2PO_4	0	81 ± 2.5	3 ± 0.1
	25	13 ± 0.9	49 ± 1.4
M9 minus NaCl	0	35 ± 3.8	53 ± 4.2
	25	11 ± 1.8	49 ± 3.8
M9 minus NH_4Cl	0	24 ± 2.5	55 ± 5.1
	25	11 ± 0.4	51 ± 3.0
M9 minus $MgSO_4$	0	37 ± 17.3	47 ±17.1
	25	12 ± 2.0	48 ± 2.2
M9 minus $CaCl_2$	0	32 ± 2.2	53 ± 0.1
	25	13 ± 0.5	49 ± 3.3
M9 minus glucose	0	24 ± 3.1	55 ± 3.7
	25	12 ± 3.7	47 ± 2.5
M9 minus phosphates	0	83 ± 4.9	3 ± 0.3
	25	52 ± 1.6	2.4 ± 0.1

S3.4 Butyraldehyde production time-course

Table S9: Time-course of butyraldehyde production by *E. coli* KS8p3

E. coli Strain	Culture Medium	Time / h	Butyraldehyde / mM	Titre / mg/L
None	H_2O	24	0	0
	M9	24	0	0
	TB + 2% w/v D-glucose	24	0	0
KS8p3		0	0	0
		1	0.12	8.7
		2	0.31	22.4
		3	0.82	59.1
		4	1.2	86.5
		18	3.7	267
		20	4.2	303
		22	4.1	296
		24	3.9	281
		26	3.8	274
		44	2.9	209
		48	2.3	166

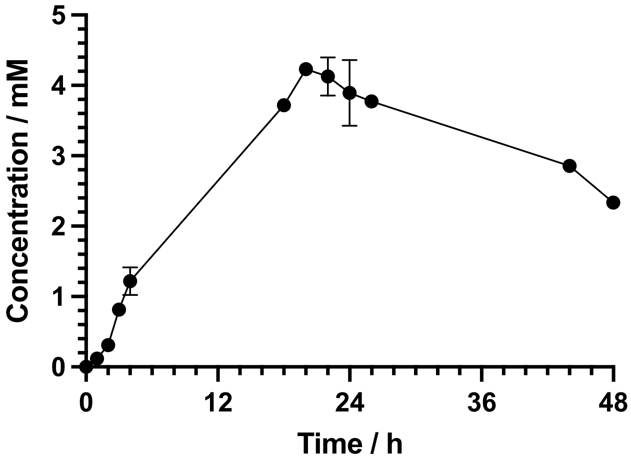

Figure S6: Time-course of butyraldehyde production by *E. coli* KS8p3

S3.5 Biocompatible α-methylenation of butyraldehyde

Table S10: Biocompatible α-methylenation of metabolic butyraldehyde in *E. coli* KS8p3. SD = standard deviation of experiments performed in triplicate. [a] Indicates L-Pro (5 mM) was added to sample before adding CH_2O

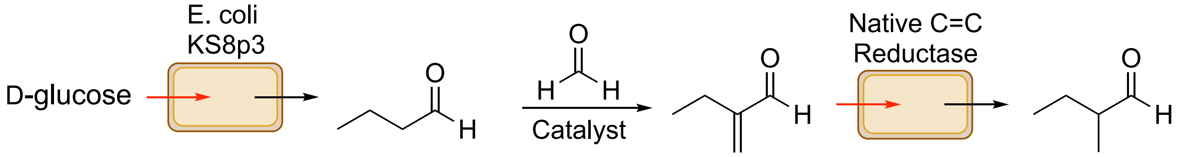

entry	CH_2O	butyraldehyde		butanol		2-EH		2-MB		2-MBA	
	mM	mM	SD	mM	SD	mM	SD	mM	SD	mM	SD
1	0	4.55	0.09	1.49	0.04	0.31	0	0	0	0	0
2	5	3.08	0.02	1.58	0	0	0	1.49	0.01	1.71	0
3	10	2.62	0.13	1.53	0.03	0.12	0.10	1.87	0.10	1.67	0.11
4	15	2.46	0.01	1.60	0.05	0	0	2.10	0.08	1.67	0.02
5	20	2.33	0.02	1.58	0.05	0	0	2.18	0.03	1.65	0.02
6	25	2.25	0.04	1.57	0.03	0	0	2.12	0.01	1.64	0
7	30	2.20	0.05	1.59	0.03	0	0	2.04	0.06	1.63	0.01
8	35	2.13	0.01	1.59	0.02	0	0	1.94	0.03	1.60	0.02
9[a]	0	5.15	0.03	1.28	0.08	0	0	0	0	0	0
10[a]	5	2.38	0.24	2.41	0.15	0	0	0.28	0.03	0.32	0.07
11[a]	10	1.30	0.07	1.64	0.06	0	0	0.72	0.12	0.1	0.03
12[a]	15	1.16	0.14	1.60	0.18	0	0	0.84	0	0	0
13[a]	20	0.78	0.21	1.46	0.08	0	0	0.68	0.12	0	0
14[a]	25	0.80	0.09	1.43	0.16	0	0	0.82	0.07	0	0
15[a]	30	0.56	0.03	1.30	0.07	0	0	0.68	0.09	0	0
16[a]	35	0.54	0.11	1.38	0.14	0	0	0.72	0.06	0	0

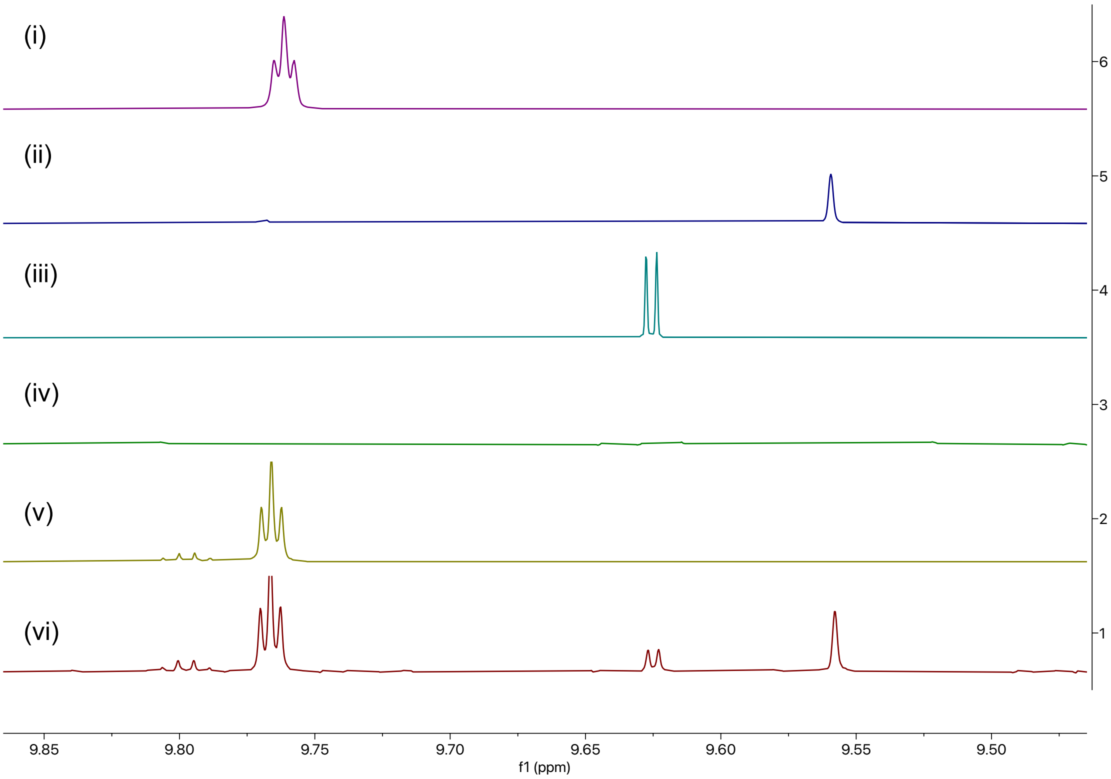

Figure S7: Stacked ^{1}H NMR spectra: (i) Butyraldehyde standard, (ii) 2-MB standard, (iii) 2-MBA standard, (iv) *E. coli* KS8p3 (uninduced), (v) *E. coli* KS8p3 (induced), (vi) *E. coli* KS8p3 (induced + formaldehyde).

S3.6 Biocompatible formal α-methylation of butyraldehyde

Table S11: Taurolidine-mediated methylenation reaction of metabolic butyraldehyde in *E. coli* KS8p3. SD = Standard deviation of experiments performed in triplicate.

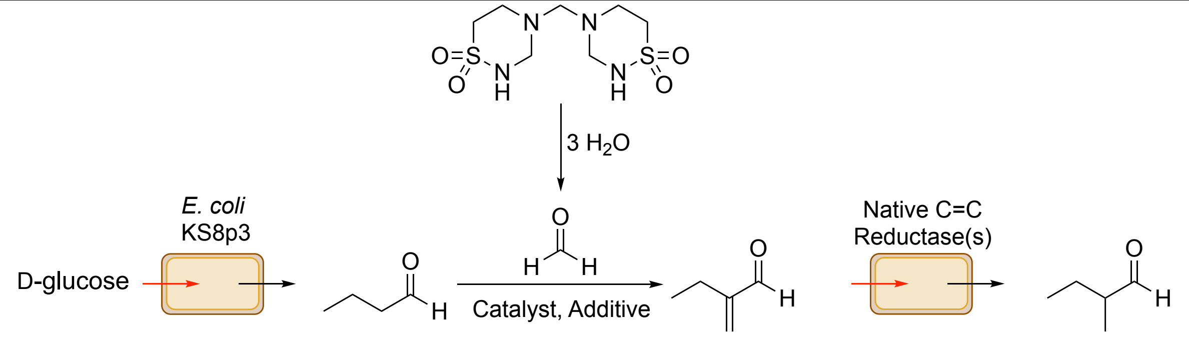

Entry	Additives		Butyraldehyde		Butanol		2-EH		2-MB		2-MBA	
		**mM**	**mM**	**SD**	**mM**	**SD**	**mM**	**SD**	**mM**	**SD**	**mM**	**SD**
1	-	-	3.74	0.13	1.34	0.12	0.17	0.01	0	0	0	0
2	Taurolidine	1	2.17	0.85	1.34	0.34	0.02	0.03	0	0	0.04	0
3	Taurolidine	5	0	0	0.54	0.06	0	0	0	0	0	0
4	Taurolidine	10	0	0	0.50	0.09	0	0	0	0	0	0
5	Taurolidine<br>L-Pro	1<br>5	1.83	0.65	1.46	0.23	0	0	0	0	0.05	0
6	Taurolidine<br>L-Pro	5<br>5	0	0	0.57	0.08	0	0	0	0	0	0
7	Taurolidine<br>L-Pro	10<br>5	0	0	0.51	0.01	0.02	0.03	0	0	0	0

S3.7 Whole-cell bioreduction of 2-MB by *E. coli* strains

Table S12: Whole-cell biotransformation of 2-MB or 2-MBA by *E. coli* BW5113, KS1p3 and KS8p3.

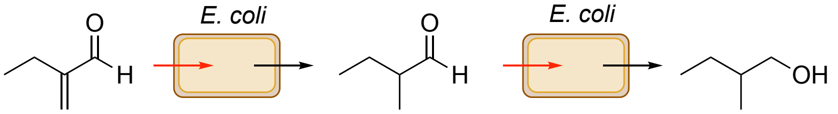

Entry	*E. coli* strain	Substrate	mM	OD_{600}	2-MB	2-MBA	2-MBO
1			2		81	19	0
2	BW25113		5		96	4	0
3			10		99	1	0
4			2		87	13	0
5	KS1p3	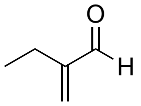	5		96	4	0
6			10		99	1	0
7			2		61	39	0
8	KS8p3		5		76	24	0
9			10	0.5	91	9	0
10			2			0	100
11	BW25113		5			0	100
12			10			28	72
13			2			0	100
14	KS1p3	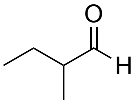	5		-	1	99
15			10			21	79
16			2			0	100
17	KS8p3		5			17	83
18			10			34	66
19				5	0	15	85
20		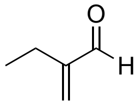		10	0	5	95
21	KS8p3		2	50	0	<1	>99
22				5		10	90
23		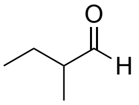		10	-	5	95
24				50		2	98

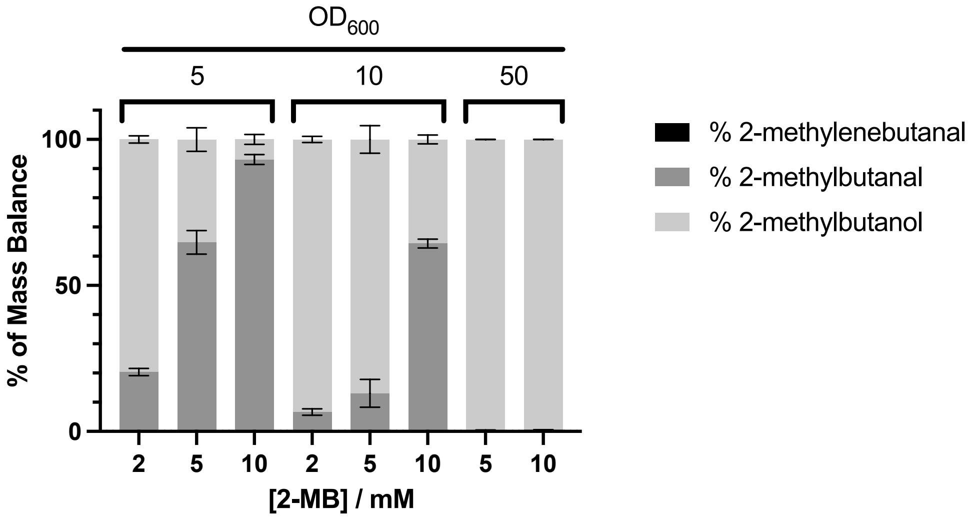

Figure S8: Variation of cell density and loading of 2-MB for whole-cell bioreduction using *E. coli* KS8p3. Error bars represent the standard deviation of triplicate samples.

Table S13: Varying cell density and substrate loading for whole-cell biotransformation of 2-MB using *E. coli*.

Entry	*E. coli* Strain	Substrate		OD_{600}	% of Mass Recovered		
			mM		2-MB	2-MBA	2-MBO
1	KS8p3	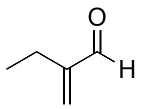	2	5	0	20	80
2			5		0	65	35
3			10		0	93	7
4			2	10	0	7	93
5			5		0	13	87
6			10		0	64	46
7			5	50	0	<1	>99
8			10		0	<1	>99
9	KS1p3		10	5	0	33	67
10				10	0	15	85
11	BW25113			5	0	<1	>99
12				10	0	<1	>99

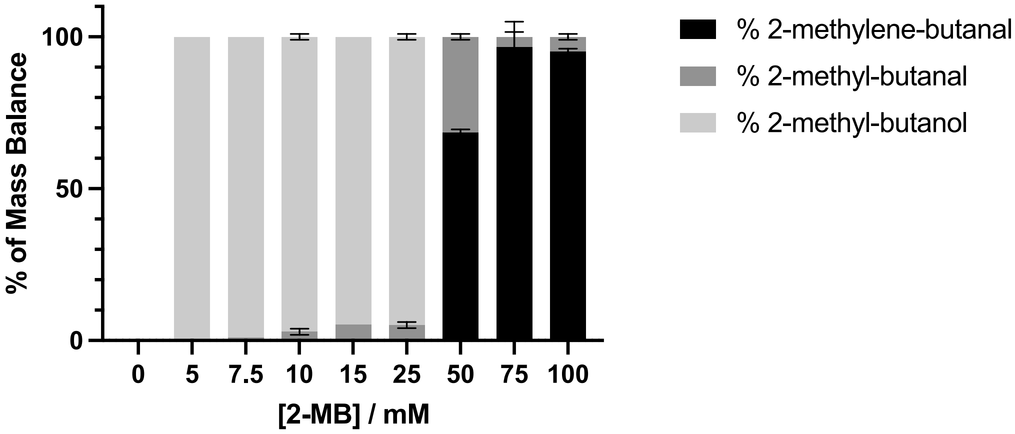

Figure S9: Increasing substrate loading for the of whole-cell bioreduction of 2-MB. Error bars represent the standard deviation of triplicate samples.

Table S14: Increasing substrate loading in the of whole-cell reduction of 2-MB

**Entry**	***E. coli* Strain**	**Substrate**		**OD_{600}**	**% of Mass Recovered**		
			**mM**		**2-MB**	**2-MBA**	**2-MBO**
1			0		0	0	0
2			5		0	<1	>99
3			7.5		0	1	99
4			10		0	3	97
5	KS8p3	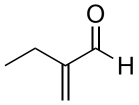	15	50	0	5	95
6			25		0	5	95
7			50		69	31	0
8			75		97	3	0
9			100		95	5	0

Table S15: Sequence alignment results from *Gluconobacter oxydans* enoate reductase (WP_011252080.1) aligned to the reference genome of *E. coli* MG1655 using NCBI BLAST blastp

Hit #	Description	Query Cover	E Value	Identity	Accession No.	Gene
1	N-ethylmaleimide reductase [*Enterobacteriaceae*]	97%	5e-86	44%	WP_000093589.1	*nemA*
2	NADPH-dependent 2,4-dienoyl-CoA reductase [*Escherichia coli*]	94%	8e-27	29%	WP_166695766.1	*fadH*

Table S16: Other alkene reductases in *E. coli* with substrate similarity to 2-MB

Entry	Gene	Enzyme Name	Known/Native Substrate(s)
1	*frdB*	NADH-dependent Fumarate reductase	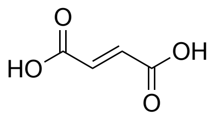
2	*curA*	NADH-dependent Curcumin reductase	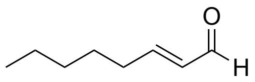 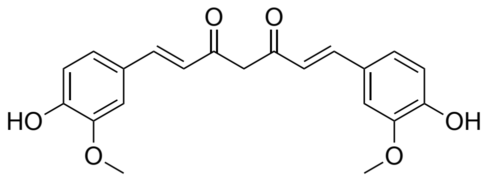

S3.8 Mosher ester analysis of bio-derived 2-MBO

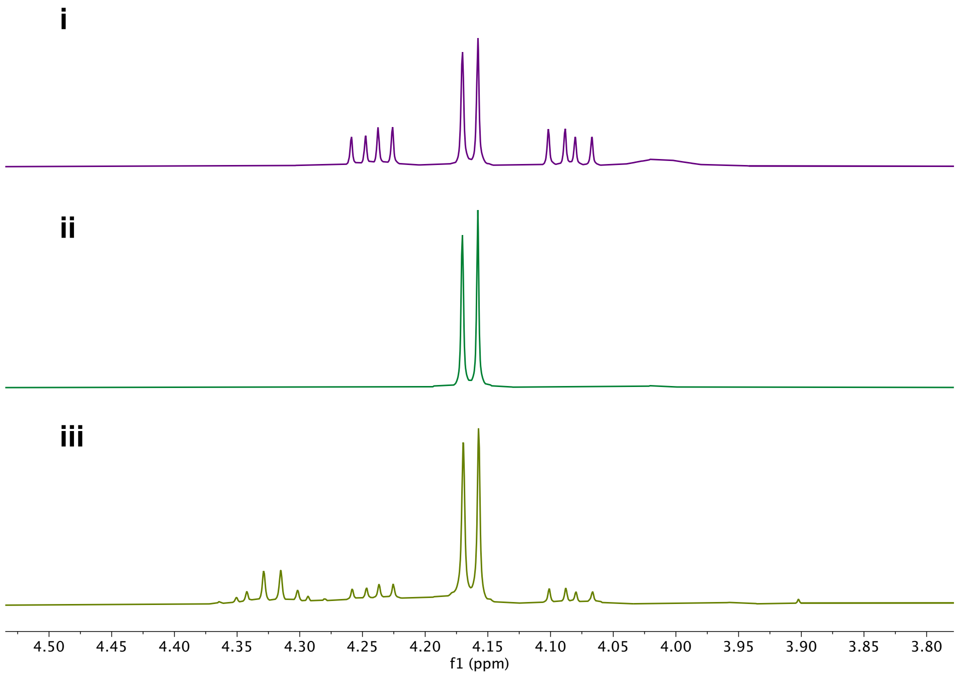

Figure S10: ^{1}H NMR spectra stack of Mosher esters of 2-MBO derived from (i) Commercial standard of rac-2-MBO (ii) Commercial standard of (S)-2-MBO (iii) Bio-derived 2-MBO

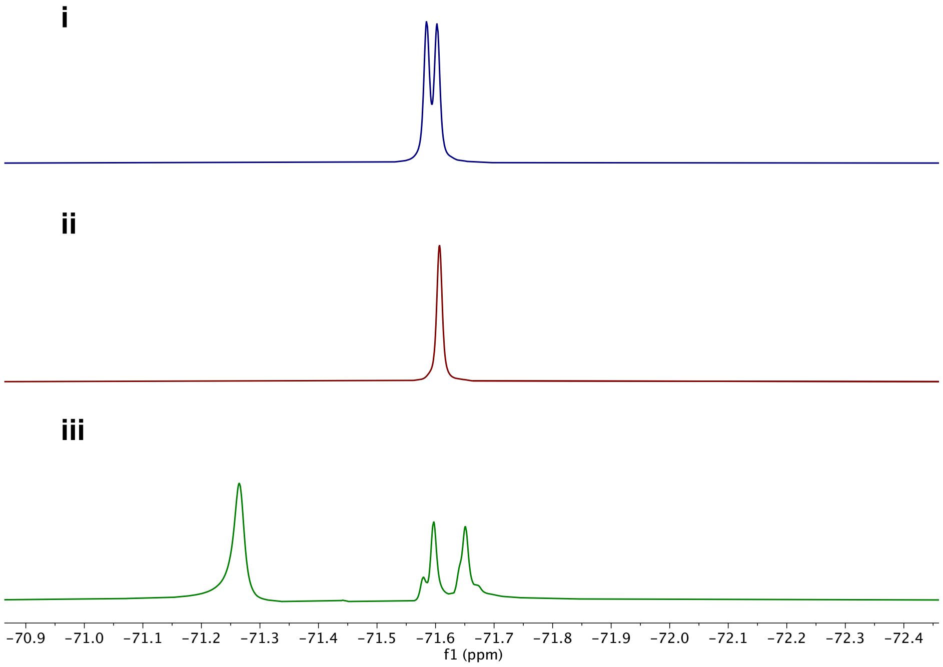

Figure S11: ^{19}F NMR spectra stack of Mosher esters of 2-MBO derived from (i) Commercial standard of rac-2-MBO (ii) Commercial standard of (S)-2-MBO (iii) Bio-derived 2-MBO

S4 References

[1] J. T. Ku, W. Simanjuntak, E. I. Lan, Biotechnol. Biofuels **2017**, 10, 291.
[2] R. C. Wen, C. R. Shen, Biotechnol. Biofuels **2016**, 9, 267.
[3] H. Oikawa, I. Matsuda, T. Kagawa, A. Ichihara, K. Kohmoto, Tetrahedron. **1994**, 50, 13347.
